# Dynamic glucocorticoid-dependent regulation of *Sgk1* expression in oligodendrocytes of adult male rat brain by acute stress and time of day

**DOI:** 10.1371/journal.pone.0175075

**Published:** 2017-04-04

**Authors:** Laura R. Hinds, Lauren E. Chun, Elizabeth R. Woodruff, Jennifer A. Christensen, Matthew J. Hartsock, Robert L. Spencer

**Affiliations:** Department of Psychology and Neuroscience, University of Colorado Boulder, Boulder, Colorado, United States of America; Technion Israel Institute of Technology, ISRAEL

## Abstract

Recent studies support plasticity in adult brain white matter structure and myelination in response to various experiential factors. One possible contributor to this plasticity may be activity-dependent modulation of serum- and glucocorticoid-inducible kinase 1 (*Sgk1*) expression in oligodendrocytes. We examined whether *Sgk1* expression in adult rat brain white matter is increased by acute stress-induced elevations in endogenous corticosterone and whether it fluctuates with diurnal variations in corticosterone. We observed rapid increases (within 30 min) in *Sgk1* mRNA in the corpus callosum in response to acute stress, as well as large increases at the beginning of the rat’s active period (the time of peak corticosterone secretion). These increases were absent in adrenalectomized rats. Corticosterone treatment of adrenalectomized rats also rapidly increased corpus callosum Sgk1 mRNA. The majority of *Sgk1* mRNA in corpus callosum was co-localized with myelin basic protein mRNA, suggesting that mature oligodendrocytes respond dynamically to acute stress and circadian rhythms. The regulation of *Sgk1* expression by acute stress and time of day was selective for white matter, with limited alteration of *Sgk1* expression by these factors in hippocampus and somatosensory cortex. These results indicate a unique sensitivity of oligodendrocyte *Sgk1* expression to activity-dependent fluctuations in corticosterone hormone secretion, and raises the prospect that hypothalamic-pituitary-adrenal axis dysregulation or glucocorticoid pharmacotherapy may compromise the normal activity-dependent interactions between oligodendrocytes and neurons.

## Introduction

Myelination within the adult brain is modulated in response to various experiential events including new training on motor and cognitive tasks [1]. Activity-dependent modulation of oligodendrocyte function is likely to underlie this plasticity in myelination [2,3], however, the molecular and cellular features of this process have yet to be established. One potential contributor to oligodendrocyte functional plasticity is the intracellular signaling molecule, serum- and glucocorticoid-inducible kinase (SGK1). *Sgk1* gene expression is rapidly upregulated in adult rat and mouse brain white matter after acute stress [4].

The *Sgk1* gene was first identified in a rat mammary tumor cell line as a result of a differential gene expression screen for short-term responses to glucocorticoids [5]. Subsequently, the rapid glucocorticoid-dependent increase of *Sgk1* mRNA has been identified in a variety of cell types [6–10], and a parallel increase in SGK1 protein levels has been observed [5]. Glucocorticoid induction of *Sgk1* mRNA persists in the face of protein synthesis inhibitors [5,7], and this induction depends on glucocorticoid receptor interaction with a glucocorticoid response element (GRE) associated with the *Sgk1* gene [5,6]. Several in vivo studies have also shown that acute glucocorticoid treatment produces an increase of *Sgk1* mRNA (within 1 hr) in white matter of rat and mouse brain [4,11,12].

In this study we explored the relationship between activity-dependent modulation of oligodendrocyte function and *Sgk1* expression by addressing 3 questions. First, are daily events that are accompanied by elevated endogenous glucocorticoid (corticosterone) hormone levels, such as moderate acute psychological stress and circadian activity, sufficient to increase *Sgk1* expression in white matter? Second, is *Sgk1* expression localized to oligodendrocytes? It is possible that the white matter expression of *Sgk1* observed in other studies reflects localization of *Sgk1* mRNA within myelinated axons [13]. Third, is experiential alteration of *Sgk1* expression in white matter paralleled by changes in neuronal *Sgk1* expression? There is high constitutive *Sgk1* mRNA levels in the CA3 region of rat hippocampus, and lower levels in other hippocampal subregions and neocortex [11,14]. Although hippocampal and neocortical *Sgk1* expression is modulated by a variety of conditions, such as transient global ischemia, hyperactivity or intracranial self-stimulation [15], no previous reports examined whether *Sgk1* expression varies in these brain regions with moderate acute psychological stress or in a diurnal fashion. We found a large increase in Sgk1 mRNA within corpus callosum in response to the corticosterone elevation associated with acute stress and circadian activity. Sgk1 mRNA in corpus callosum was localized to myelin basic protein expressing cells (oligodendrocytes). Finally, there was a much more limited effect of acute stress and time of day on Sgk1 expression in hippocampus and a region of neocortex (somatosensory cortex).

## Materials and methods

### Subjects

Male Sprague-Dawley rats (250-280g) were obtained from a commercial vendor (Harlan Laboratories, Indianapolis, IN) and were pair housed (polycarbonate tubs, 47 cm × 23 cm × 20 cm) after arrival at the University of Colorado Boulder animal facility. Rats were maintained on a 12 h light/dark cycle and given food (Teklad Rodent Diet 8640; Harlan) and either tap water (Sham rats) or 0.9% saline ± corticosterone (ADX rats) *ad libitum*. Experimental time of day for each rat is expressed as zeitgeber time (ZT), which is the time (h) after the onset of the rat’s daily light phase. All experiments were conducted in accordance with ethical treatment of animals and were approved by the University of Colorado Institutional Animal Care and Use Committee.

### Experimental procedures

#### Experiment 1: Effect of acute stress, time of day and adrenal status on *Sgk1* mRNA

The effect of adrenal status and time of day on *Sgk1* mRNA response to acute stress (30 min restraint) was examined in a 2 X 2 X 2 factorial design (N = 50; n = 6–7): adrenal status (Sham or ADX) X time of day (ZT4 or ZT16) X stress (home cage or 30 min restraint). Two weeks after arrival at the University of Colorado Boulder animal facility rats were given adrenalectomy (ADX) or control surgery (SHAM). On the test day (1 week after surgery) rats were either challenged with 30 min of restraint in Plexiglas tubes (6.3 cm diameter, 14 cm length) or left undisturbed in their home cage at ZT3.5 or ZT15.5. Rats were then killed by guillotine decapitation at ZT4 or ZT16. Procedures during the dark phase were performed under red light conditions. Brains were removed immediately, rapidly frozen in isopentane chilled with dry ice to ~-20°C and stored at -70°C. Trunk blood was also collected and plasma was stored at -70°C.

#### Experiment 2: *Sgk1* mRNA in adrenal-intact and ADX rats at ZT0, ZT6, ZT12, and ZT18

The effects of adrenal status and time of day on non-stressed rat *Sgk1* mRNA levels were further examined in a 2 X 4 factorial design (N = 49; n = 6–7): adrenal status (Sham or ADX) X time of day (ZT0, ZT6, ZT12 or ZT18). On the test day (12 days after surgery) rats were left undisturbed in their home cage until they were killed by guillotine decapitation. Rats that were killed at ZT0 or ZT12 were killed 15 min before the light:dark transition to rule out any acute effect of the sudden change in light condition. Other measures (clock gene expression) on tissue from these rats have been reported previously [16], and as part of that study’s objectives each of these rats were given a daily vehicle (60% sterile saline, 30% propylene glycol, 10% ethanol) injection (1 ml/kg, i.p.) at ZT1 for 10 days prior to the test day. Red light conditions were used for procedures performed during the dark phase, and brains and trunk blood were collected and stored as described above for Experiment 1.

#### Experiment 3: Effect of acute systemic corticosterone (CORT) on *Sgk1* mRNA at ZT4 and ZT16 in ADX rats

To examine the effect of CORT on *Sgk1* expression during the rat’s active and inactive phase, a 2 X 3 factorial design was used (N = 35; n = 5–6): time of day (ZT4 or ZT16) X injection condition (no injection, vehicle injection, or CORT injection). Rats were acclimated to lighting conditions at the University of Colorado Boulder animal facility for two weeks before undergoing ADX surgery during the second half of the light phase. For three days after surgery, CORT (25 μg/ml; Steraloids Inc., Newport, RI) was replaced in the post-operative drinking water (tap water containing 0.1% ethanol, 0.9% saline, and 0.04% ibuprofen (Pfizer, Madison, NJ)) during the first two hours of the dark phase in order to preserve diurnal variations in baseline CORT levels [17]. On the third day after surgery, rats received no injection, vehicle injection (10% ethanol, 30% propylene glycol, 60% sterile saline, 1 ml/kg i.p.), or CORT injection (5 mg/kg, i.p.) at ZT3.5 or ZT15.5 and were killed 30 min later by guillotine decapitation. Red light conditions were used for procedures performed during the dark phase, and brains and trunk blood were collected and stored as described above for Experiment 1.

### Adrenalectomy surgery

Rats were deeply anesthetized with halothane gas delivered by a vaporizer (Vetequip; Pleasanton, CA). Small bilateral incisions were made through the dorsolateral skin and peritoneal wall near the kidney. The adrenal glands were carefully grasped by an intestinal tissue forceps and then excised from the surrounding fat tissue with scissors, thereby allowing removal with minimal disturbance to other internal organs. Sham surgery followed the same procedure, but the adrenal glands were not removed.

### Plasma corticosterone (CORT)

Plasma CORT was measured using a commercial enzyme-linked immunosorbant assay for CORT (Arbor Assays, Ann Arbor, MI). Instead of use of the steroid displacement reagent provided by the kit, samples were instead diluted 1:50 in assay buffer and heated to 65°C for one hour in order to completely inactivate corticosteroid binding globulin [17]. Intra-assay coefficients of variation were less than 10% for each experiment.

### In situ hybridization for *Sgk1* and *Mbp* mRNA

Exonic portions of rat serum- and glucocorticoid- inducible kinase (*Sgk1*; Genebank accession no. NM_001193568.1: nuclear transcript 912–1619) and myelin basic protein (*Mbp*; Genebank accession no. NM_001025291.1: nuclear transcript 82–569) were isolated by RT-PCR and cloned into vectors suitable for subsequent generation of riboprobes. Briefly, using whole rat brain, total RNA was isolated (SV Total RNA extraction system; Promega) and cDNA was generated with the Superscript III kit (Life Technologies). Using the cDNA as a template, PCR fragments for *Sgk1* were amplified with GoTaq Hot Start polymerase (Promega) and the resulting purified amplicon cloned using Strataclone PCR Cloning Kit (Stratagene). For *Mbp* fragment cloning, tissue with a high proportion of white matter was isolated using the Trizol method (Life Technologies) then cDNA generated with the Superscript III kit was used as a template for PCR with OneTaq polymerase (New England Biolabs) and inserted into a PCRII-TOPO vector (Life Technologies). Confirmation of desired product and orientation for both gene products were validated by DNA sequencing (Genewiz). In situ hybridization using antisense riboprobes yielded autoradiograms with strong specific signal in expected brain regions, whereas sense riboprobes yielded autoradiograms with no specific signal in any brain region examined (data not shown).

Coronal slices (12 μm) from experimental brains were sectioned via cryostat through a portion of the dorsal hippocampus (~-3.0 to -4.0 Bregma), thaw-mounted onto charged slides (Superfrost Plus, VWR) and stored at -70°C. In situ hybridization was performed similar to previous descriptions [18]. Briefly, slides were prepared for hybridization by fixation in a buffered 4% paraformaldehyde solution for 15 minutes followed by a sodium citrate buffer wash (0.3M NaCl, 0.03M sodium citrate), acetylated for 10 minutes (0.1M triethanolamine, 0.25% acetic anhydride, pH 8), rinsed in RNAse-free water and dehydrated in a series of increasing ethanol solutions. Slides were air-dried at room temperature. A hybridization buffer containing 1.5 x 10^6^ cpm of 35-S labeled UTP incorporated into anti-sense *Sgk1* or *Mbp* fragments was applied to each slide and cover slipped. Slides were then incubated at 54°C for 16 hours in 50% formaldehyde/50% water humidified chambers. Slides were then washed with sodium citrate buffer and treated with 200 μg RNAse A (Sigma) followed by washes of increasingly diluted sodium citrate buffer. Slides were subjected to a final high stringency wash (65°C, 1 hr) in dilute sodium citrate buffer and then dehydrated in ethanol solutions and air dried. Slides were exposed to x-ray film (Kodak BioMax MR) for ~1–2 weeks.

For double-label (*Sgk1* mRNA and *Mbp* mRNA) fluorescent-tagged in-situ hybridization, slides were treated as above except either dioxygenin-labeled UTP (for *Sgk1*) or fluorescein-labeled-UTP (for *Mbp*) was incorporated into the anti-sense riboprobe. Initial fixation, hybridization and post-hybridization procedures were the same as above until completion of the high stringency wash, slides were then transferred to 0.05M sodium phosphate buffered saline (PBS) and stored at 4°C overnight. The next day, slides were washed in PBS and then endogenous peroxidase was quenched by incubation in phosphate-buffered 2% hydrogen peroxide (30 min). Slides were then washed in 0.1M Tris buffered saline (TBS) with 0.05% Tween-20 (TBS-T) followed by 0.5% Blocking Reagent (Perkin-Elmer) in TBS. For detection of *Sgk1* mRNA, slides were exposed to horseradish-peroxidase linked anti-dioxygenin antibody (Roche) at 1:750 in 0.5% Blocking Reagent for 30 minutes, then washed with 0.1M TBS. Amplification of the signal with a fluorescent marker was performed using the Cy3 Tyramide Amplification Reagent (Perkin Elmer) according to manufacturer’s directions. Slides were washed with 0.1M TBS-T and the process was repeated for detecting the *Mbp* mRNA using anti-fluorescein-HRP (1:100, Perkin Elmer) and the Fluorescein Tyramide Amplification Reagent (Perkin Elmer). Slides were rinsed with PBS, incubated with DAPI nuclear stain at 1:30,000 (Fisher cat # 50850585) and then rinsed again. Wet slides were coverslipped with mounting media (Fluoromount; Southern Biotech).

### Autoradiographic densitometry

**Fig 1:**
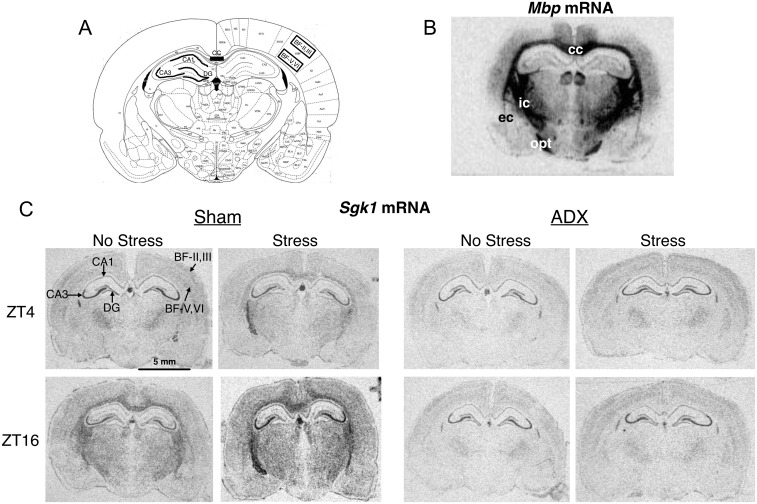
Representative autoradiograms of *Mbp* mRNA and experiment 1 *Sgk1* mRNA. (A) *Mbp* mRNA is heavily expressed in the major white matter tracts, including the corpus callosum (cc), internal capsule (ic), external capsule (ec) and optic tract (opt). (B) Under no stress conditions at ZT4 there was very little *Sgk1* mRNA levels in white matter, but high levels in the CA3 region of the hippocampus, and moderate levels in the CA1 and dentate gyrus (DG) subregions of the hippocampus, and the inner (BF-V, VI) and outer (BF-II, III) layers of the barrel fields of the neocortex. Note the dramatic upregulation of *Sgk1* mRNA visible in white matter after acute stress and at ZT16 of adrenal-intact (Sham) rats but not ADX rats.

In situ hybridization autoradiographs were digitized using Scion Image (Scion Corporation) in a single session per experiment to prevent image capture session artifacts during analysis. An individual blind to treatment group assignments used ImageJ (NIH) to measure average uncalibrated optical density (OD) values for regions of interest (Fig 1) corresponding to brain regions defined in the rat brain atlas by Paxinos and Watson [19]. For each region of interest, measurements were performed on both hemispheres of 4–6 sections per brain and then averaged.

### Statistical analyses

Data were analyzed by multifactor analysis of variance (ANOVA), and when appropriate followed by Fisher’s Least Significant Difference (FLSD) post-hoc test (IBM SPSS, version 23.0 for MacIntosh OS, Chicago, IL). An alpha level of p < .05 was adopted a priori for all analyses. Data presented in graphs are means ± SEM.

## Results

### Experiment 1: Effect of acute stress, time of day and adrenal status on *Sgk1* mRNA

Using radioactive in situ hybridization we characterized the expression of *Sgk1* mRNA in corpus callosum, hippocampal subregions, and neocortex (somatosensory cortex—barrel fields) in rats subjected to 30-minute restraint stress at times of day when CORT is typically low (ZT4) or typically high (ZT16). Adrenal glands were removed from one set of rats for each time of day and stress condition in order to determine whether any effect of acute stress or time of day on *Sgk1* expression depended on the presence of endogenous CORT.

#### Relationship between *Sgk1* mRNA in corpus callosum and CORT levels

**Fig 2:**
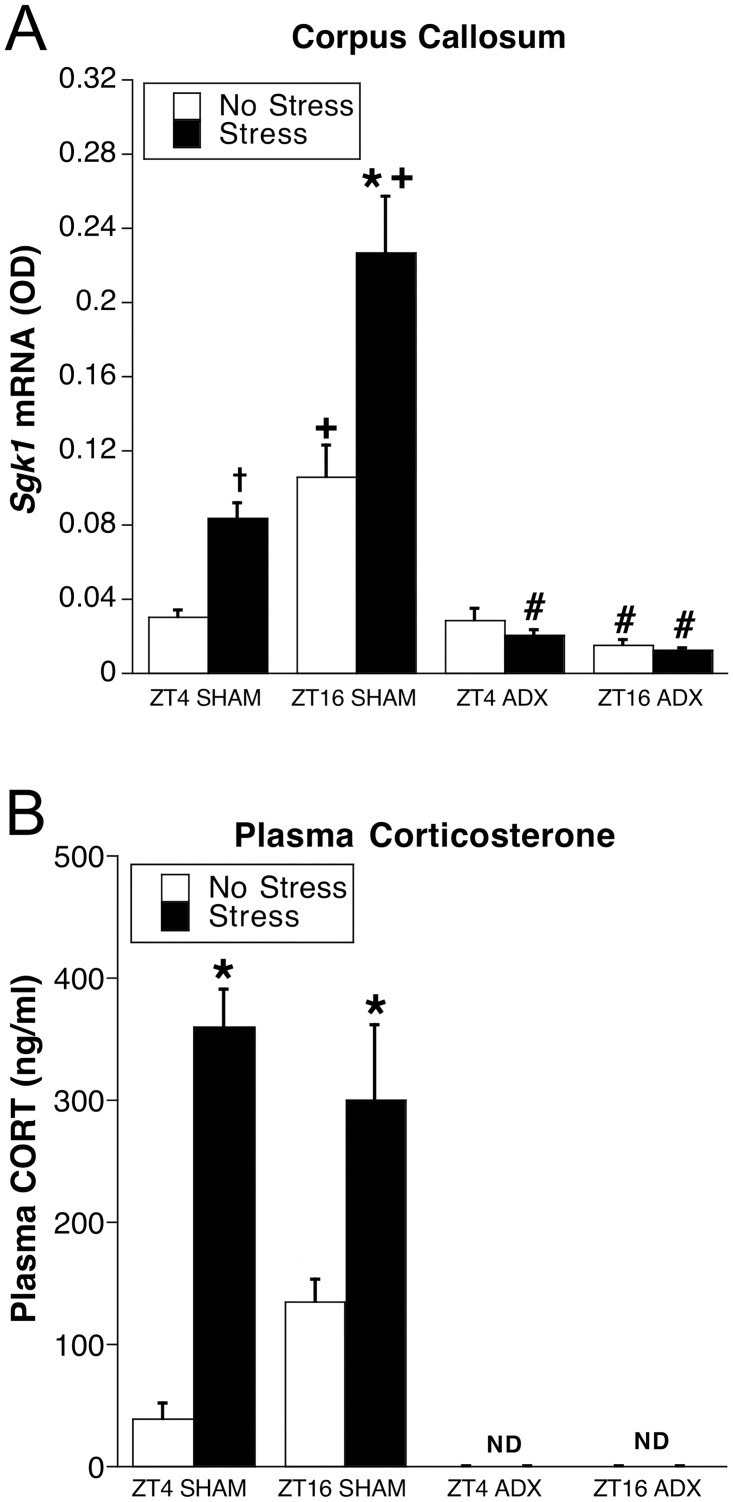
Experiment 1: *Sgk1* mRNA in corpus callosum (A) and plasma CORT (B). Adrenal-intact (SHAM) or ADX rats were killed at either ZT4 or ZT16 after 30 min of restraint (Stress) or no stress (n = 6–7). *p < 0.05 compared to no stress group at same ZT and within same adrenal status; +p < 0.05 compared to ZT4 within same stress condition and adrenal status; #p < 0.05 compared to SHAM rats at same ZT and within same stress condition (FLSD). ND = not detectable.

Visual inspection of representative autoradiographic coronal brain images of *Sgk1* mRNA expression across the eight treatment groups shows a dramatic effect of acute stress, time of day and adrenal status on *Sgk1* mRNA levels within the major white matter structures of the corpus callosum, internal capsule and external capsule (compare *Sgk1* mRNA regional expression with that of the oligodendrocyte marker *Mbp* mRNA, Fig 1). Given the discrete boundary of the corpus callosum with surrounding gray matter, we chose the corpus callosum as a region of interest (ROI) for quantitative analysis of treatment effects on white matter *Sgk1* mRNA. There were very low *Sgk1* mRNA levels in the corpus callosum of unstressed rats at ZT4, and a large diurnal increase in adrenal-intact rats at ZT16 (Figs 1 and 2). Restraint produced a significant increase in *Sgk1* mRNA at both times of day in adrenal-intact rats, however, the stress-induced *Sgk1* mRNA levels at ZT4 were not as high as the basal levels present at ZT16. Consequently, stress-induced *Sgk1* mRNA levels were significantly higher at ZT16 than at ZT4, although the proportional increase relative to basal levels at ZT16 (214%) was somewhat less than at ZT4 (280%). All ADX rats had low *Sgk1* mRNA levels in the corpus callosum, regardless of time of day or exposure to restraint (Figs 1 and 2). In support of this pattern of results there was an overall significant effect of time of day (F_1,40_ = 26.8, p < .01), stress (F_1,40_ = 18.4, p < .01), and adrenal status (F_1,40_ = 94.7, p < .01). There was also a significant time of day X adrenal status interaction (F_1,40_ = 39.5, p < .01) and a stress X adrenal status interaction (F_1, 40_ = 23.7, p < .01).

Expression of *Sgk1* mRNA in corpus callosum was related to, but not perfectly mirrored by, CORT levels in each treatment group (Fig 2). As expected, basal CORT levels were low at ZT4 and significantly higher at ZT16 (Fig 2b). Restraint resulted in high CORT levels of adrenal-intact rats that, in contrast to *Sgk1* mRNA levels, were similar at both times of day. CORT levels were below the reliable assay detection limit in ADX rats.

#### *Sgk1* mRNA in hippocampus

**Fig 3:**
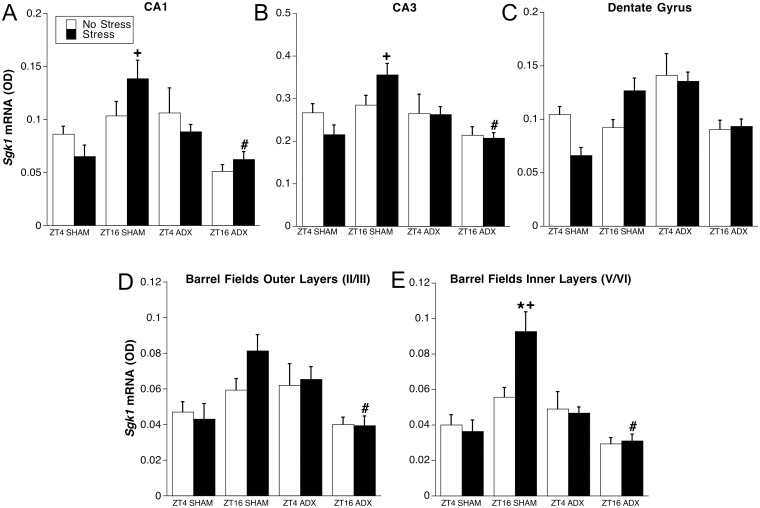
Experiment 1: *Sgk1* mRNA in hippocampus (A-C) and barrel fields (D and E) of somatosensory cortex. Adrenal-intact (SHAM) or ADX rats were killed at either ZT4 or ZT16 after 30 min of restraint (Stress) or no stress (n = 6–7). *p < 0.05 compared to no stress group at same ZT and within same adrenal status; +p < 0.05 compared to ZT4 within same stress condition and adrenal status; #p < 0.05 compared to SHAM rats at same ZT and within same stress condition (FLSD).

Visual inspection of representative autoradiographic coronal brain images of *Sgk1* mRNA expression (Fig 1B) shows for all treatment groups high level expression in the principal cell layer of the CA3 subregion and lesser but distinct expression in the principal cell layers of the CA1 and dentate gyrus subregions. In contrast to the corpus callosum, there was a much more limited effect of the various treatments on *Sgk1* mRNA levels in hippocampus (Fig 3A–3C). Across all three hippocampal subregions there was no main effect of stress or time of day. There was an overall significant effect of adrenal status in the CA1 (F_1,40_ = 4.2, p = .048) and CA3 (F_1,40_ = 4.4, p = .04) subregions with lower mean *Sgk1* mRNA levels in ADX rats compared to adrenal intact rats. In each hippocampal subregion there was a significant time of day by adrenal status interaction (CA1: F_1,40_ = 16.7, p < .001; CA3: F_1,40_ = 10.4, p = .002; dentate gyrus: F_1,40_ = 9.3, p = .004). Post hoc tests indicate that this interaction is primarily due to significantly higher *Skg1* mRNA levels present in adrenal-intact rats stressed at ZT16 compared to ZT4, whereas this time of day difference shows an opposite pattern in ADX rats.

#### *Sgk1* mRNA in barrel fields cortex

Visual inspection of representative autoradiographic coronal brain images of *Sgk1* mRNA expression (Fig 1B) shows for all treatment groups a moderate level of expression in neocortex with a somewhat greater expression in the outer (layers II and III) and inner (layers V and VI) layers than middle layer (layer IV). This pattern is especially evident in the barrel fields region of somatosensory cortex. We used the outer and inner layers of the barrel fields as regions of interest that may reflect general neocortical patterns of *Sgk1* mRNA expression under our various treatment conditions. The general pattern of treatment effects on *Sgk1* mRNA levels in the barrel fields was similar to what we observed in the hippocampus (Fig 3). There was not a main effect of stress or time of day, but in the inner layers a main effect of adrenal status (F_1,40_ = 10.3, p = .003). As was the case in each hippocampal subregion, for both the outer and inner layers of the barrel fields there was a significant time of day by adrenal status interaction (outer layers: F_1,40_ = 15.8, p < .001; inner layers: F_1,40_ = 25.26, p < .001). In the inner layers there was also a significant time of day by stress interaction (F_1,40_ = 4.44, p = .04). Similar to the hippocampus, post hoc tests indicate that these interactions are primarily due to significantly higher *Sgk1* mRNA levels present in adrenal-intact rats stressed at ZT16 compared to ZT4, whereas this time of day difference shows an opposite pattern in ADX rats.

#### Co-localization of *Sgk1* mRNA with *Mbp* mRNA in corpus callosum but not hippocampus

**Fig 4:**
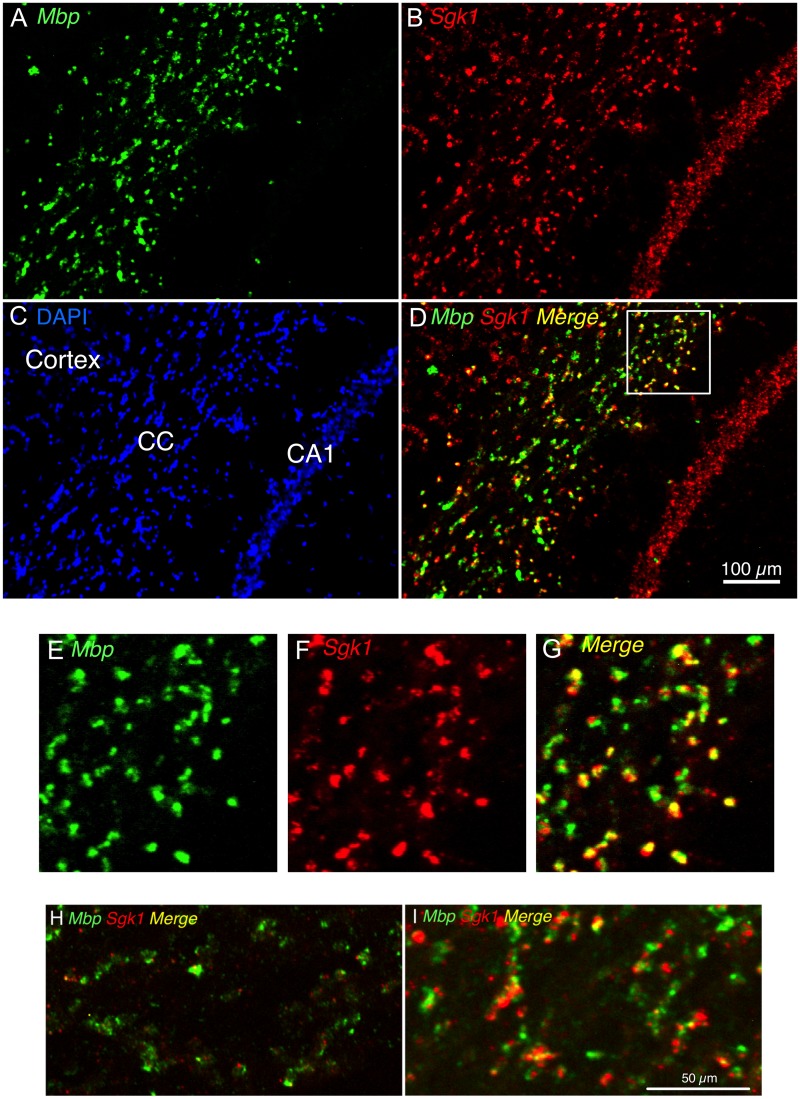
Representative photomicrograph showing *Mbp* mRNA and *Sgk1* mRNA co-localization in corpus callosum but not hippocampus. (A-D) Representative immunofluorescence image for *Mbp* mRNA (green; A), *Sgk1* mRNA (red; B) and their overlap (Merge; yellow; D) in a portion of a coronal brain section from a Sham rat that was exposed to acute stress at ZT4. (C) DAPI nuclear counterstain illustrates the anatomical subregions present: corpus callosum (CC), hippocampus (CA1) and neocortex (Cortex). (E-G) Enlargement of the portion of the image in panel D outlined within the white box.

Given the large upregulation of *Sgk1* mRNA observed in the corpus callosum after acute stress and during the dark phase we examined whether *Sgk1* mRNA was co-localized with oligodendrocytes of the corpus callosum. Using double-label fluorescent in situ hybridization for *Sgk1* mRNA and *Mbp* mRNA we found that most of the *Sgk1* mRNA present in the corpus callosum of adrenal-intact rats exposed to acute stress was co-localized with *Mbp* mRNA (Fig 4). *Sgk1* mRNA was also present in many presumed pyramidal neurons of CA1 (Fig 4B) and CA3 as well as presumed granule neurons of dentate gyrus. Most of these hippocampal cells were negative for *Mbp* mRNA, as expected (Fig 4D).

### Experiment 2: *Sgk1* mRNA in adrenal-intact and ADX rats at ZT0, ZT6, ZT12, and ZT18

**Fig 5:**
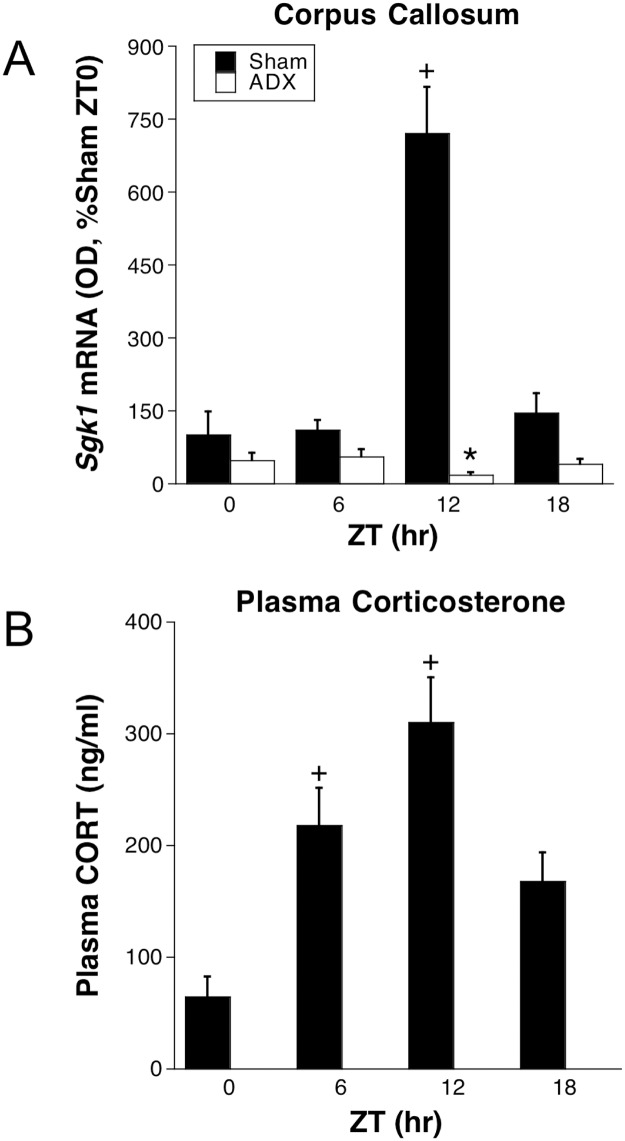
Experiment 2: *Sgk1* mRNA in corpus callosum (A) and plasma CORT (B). Adrenal-intact (SHAM) or ADX rats were killed at ZT0/24 (double-plotted), ZT6, ZT12, and ZT18 (n = 6–7). *p < 0.05 compared to Sham rats at same ZT; +p < 0.05 compared to ZT0/24 within same adrenal status (FLSD).

**Fig 6:**
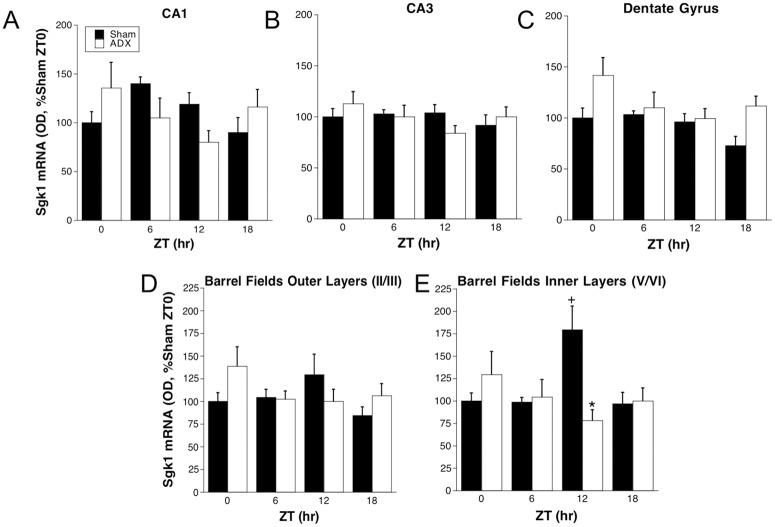
Experiment 2: *Sgk*1 mRNA in hippocampus (A-C) and barrel fields of somatosensory cortex (D-E). Adrenal-intact (SHAM) or ADX rats were killed at ZT0/24 (double-plotted), ZT6, ZT12, and ZT18 (n = 6–7). *p < 0.05 compared to Sham rats at same ZT; +p < 0.05 compared to ZT0/24 within same adrenal status (FLSD).

This experiment further examined the profile of *Sgk1* mRNA expression throughout the day of unstressed rats and its relationship to diurnal basal CORT secretion. *Sgk1* mRNA levels in the corpus callosum of adrenal-intact rats were high at ZT12, however, they were uniformly low at the other times of day (Fig 5A). Basal plasma CORT levels were also highest at ZT12, lowest at ZT0/ZT24 and intermediate at ZT6 and ZT18 (Fig 5B). In ADX rats corpus callosum *Sgk1* mRNA levels were low at all times of day. ANOVA results confirm statistical significance for this pattern of results: time of day, F_3,39_ = 24.7, p < .001; adrenal status, F_1,39_ = 60.9, p < .001; and time of day by adrenal status interaction, F_3,39_ = 30.5, p < .001. In the other brain regions examined there were no significant effects of time of day on *Sgk1* mRNA levels (Fig 6). In two of those brain regions, however, there was a significant time of day by adrenal status interaction (CA1: F_3,40_ = 2.9, p = .048; inner layers barrel fields: F_3,39_ = 5.7, p = .003). The interaction in the inner layers of the barrel fields was particularly pronounced with a pattern very similar to that observed in the corpus callosum (i.e. a significant increase in Sgk1 mRNA at ZT12 in adrenal-intact rats that was absent in ADX rats). In the dentate gyrus there was a main effect of adrenal status (F_1,40_ = 4.1, p = .049), reflected by higher levels of *Sgk1* mRNA in ADX rats at ZT18 and ZT0/24.

### Experiment 3: Effect of acute systemic (CORT) on *Sgk1* mRNA at ZT4 and ZT16 in ADX rats

In experiment 1, acute stress produced significantly higher *Sgk1* mRNA levels in the corpus callosum of adrenal-intact rats at ZT16 compared to ZT4. This time of day difference may reflect a greater sensitivity of corpus callosum *Sgk1* expression to stress-induced CORT levels at ZT16, or an additive effect of high basal and stress-induced *Sgk1* mRNA levels at ZT16. This experiment, therefore, tested the extent to which acute CORT treatment of ADX rats increases *Sgk1* expression at ZT4 and ZT16.

**Fig 7:**
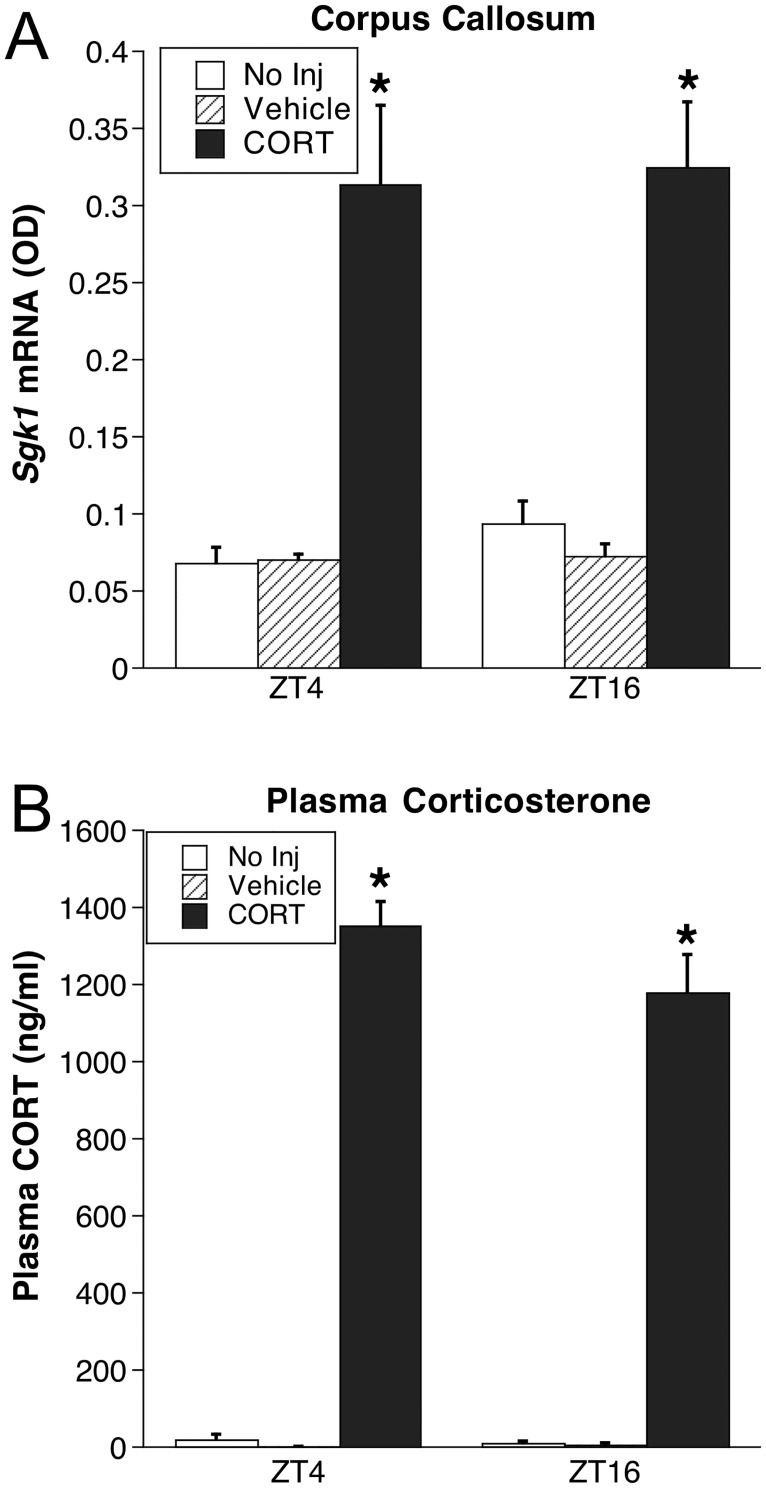
Experiment 3: *Sgk1* mRNA in corpus callosum (A) and plasma CORT (B). ADX rats were injected with CORT (5 mg/kg i.p.), vehicle, or were left undisturbed in their home cage (No inj) (n = 5–6), and were then killed 30 min later at ZT4 or ZT16. *p < 0.05 compared to No inj rats at same ZT (FLSD).

Compared to rats receiving no injection, CORT-treated rats, but not vehicle treated rats, exhibited a large increase in *Sgk1* mRNA expression in the corpus callosum (injection condition, F_1,34_ = 46.9, p < .001, followed by FLSD post hoc test, p < .05) (Fig 7). The CORT-induced increase in *Sgk1* mRNA was similar at both times of day. As expected, CORT treatment produced relatively high levels of plasma CORT 30 min after injection that were in the high physiological stress range [17], and these circulating levels were similar at both times of day.

**Fig 8:**
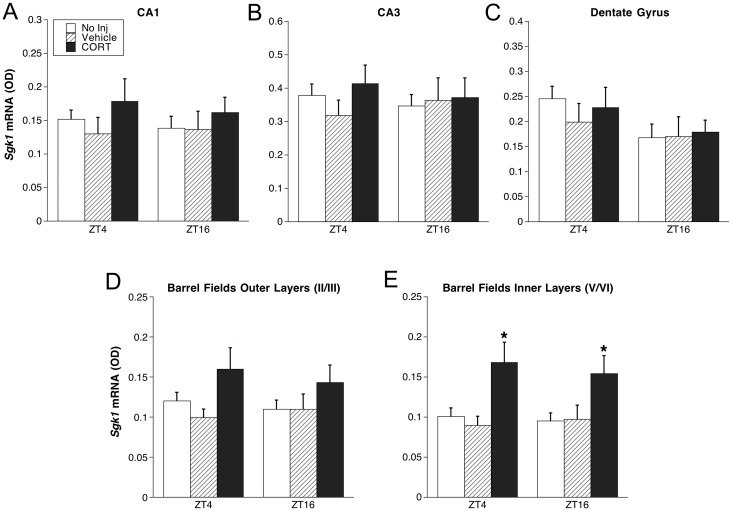
Experiment 3: *Sgk*1 mRNA in hippocampus (A-C) and barrel fields of somatosensory cortex (D-E). ADX rats were injected with CORT (5 mg/kg i.p.), vehicle, or were left undisturbed in their home cage (No inj) (n = 5–6), and were then killed 30 min later at ZT4 or ZT16. *p < 0.05 compared to No inj rats at same ZT (FLSD).

There was no effect of injection condition or time of day on *Sgk1* mRNA levels in the hippocampal subregions. However, CORT produced a moderate elevation of *Sgk1* mRNA in the barrel fields (Fig 8). Two-way ANOVA indicated a main effect of injection condition in the barrel fields outer (F_1,34_ = 3.962, p < 0.05) and inner (F_1,34 *=*_ = 9.573, p < 0.05) layers, with CORT treated rats showing elevated *Sgk1* mRNA expression relative to rats receiving no injection or vehicle injection (FLSD, p < .05).

## Discussion

We found that the expression of *Sgk1* was dynamically regulated in corpus callosum by acute stress and time of day, and that this regulation was CORT dependent. *Sgk1* mRNA within the corpus callosum was co-localized with *Mbp* mRNA, suggesting that this dynamic regulation of *Sgk1* expression occurred within oligodendrocytes. Specifically, we found in corpus callosum that *Sgk1* mRNA was dramatically upregulated by acute psychological stress (30 min restraint) and by the onset of the rat’s active circadian phase.

Our results are consistent with another report that found that a more intense acute stressor (restraint + partial water immersion) increased *Sgk1* mRNA in white matter of mouse brain [4]. In both that study and ours, the increase in *Sgk1* mRNA by acute stress was absent in ADX rats, suggesting that the acute stress effect on *Sgk1* expression depends on stress-induced elevation of CORT. Our study is the first to show that there is also a large daily upregulation of *Sgk1* mRNA in corpus callosum that peaks around the beginning of the rat’s active period. This diurnal peak in *Sgk1* mRNA coincides with the diurnal peak in basal CORT secretion (ZT12), and it was absent in ADX rats. Interestingly, we also observed a time of day difference in the relative levels of stress-induced Sgk1 mRNA in the corpus callosum, with much higher levels after acute stress at ZT16 than ZT4. One possible basis for this difference is that there is an intrinsic circadian variation in the sensitivity of *Sgk1* induction by acute CORT in oligodendrocytes. However, we found that acute CORT treatment of ADX rats produced comparable increases in *Sgk1* mRNA at both times of day. Consequently, the greater levels of stress-induced *Sgk1* mRNA at ZT16 may be due to an additive effect of stress-induced *Sgk1* expression superimposed on higher basal levels of expression at that time of day.

Although *Sgk1* was first identified in various cell lines because of its rapid induction by glucocorticoids [5,7], it is interesting to note that the dynamic in vivo CORT-dependent regulation of *Sgk1* expression in the brain is largely restricted to white matter. There is high constitutive expression of *Sgk1* mRNA in the hippocampus, especially within the CA3 subregion. The hippocampal *Sgk1* mRNA is predominantly localized within the principal cell layer of Ammon’s horn (pyramidal neurons) and dentate gyrus (granule neurons) (Figs 1 and 4), indicating that this hippocampal expression is likely neuronal. Other studies have also reported hippocampal expression of *Sgk1* mRNA, as well as expression in neocortex [11,12,14,20]. In this study we examined *Sgk1* mRNA expression in the neocortical region of the somatosensory barrel fields [21]. In contrast to our findings in corpus callosum, we observed a much more limited diurnal and acute stress modulation of *Sgk1* mRNA in hippocampus and neocortex. Only in the outer layers of the barrel fields did we observe significant upregulation of *Sgk1* mRNA by acute stress (however, only at ZT16) and an adrenal-dependent diurnal increase at ZT12. Acute CORT also produced a significant increase in *Sgk1* mRNA within the barrel fields. These results may indicate that CORT regulation of *Sgk1* expression is similar in oligodendrocytes and this cortical neuronal population. We cannot rule out the possibility, however, that there may be a greater number of oligodendrocytes intermixed in this neocortical region compared to the hippocampus. Thus, the altered *Sgk1* expression within the barrel fields may be primarily a result of gene expression changes within oligodendrocytes. It is noteworthy that we see a significant amount of *Mbp* mRNA expression within this region of neocortex (Fig 1A).

Although another study also failed to see an effect of acute stress on *Sgk1* mRNA in hippocampus [4], two more recent studies observed a significant increase of Sgk1 expression after swim stress (but not restraint) [22,23]. This increase was much more evident when examining Sgk1 hnRNA levels rather than Sgk1 mRNA levels, likely due to the increased sensitivity that the hnRNA measure provides for detecting relatively rapid but transient changes in gene expression [22]. Whether glucocorticoid treatment induces *Sgk1* expression in the hippocampus also varies across studies. Consistent with our results, one other study did not see an effect of acute CORT treatment on rat hippocampal *Sgk1* mRNA [11], whereas a separate study observed some increase in CA1, but not CA3 [12]. Glucocorticoid treatment of a human hippocampal progenitor cell line produced an increase in *Sgk1* mRNA [24]. The limited effect of acute stress and time of day on *Sgk1* mRNA in the hippocampus may be due to the relatively high constitutive expression of the gene relative to that observed in the corpus callosum, which has very low expression in the absence of stress at ZT4. There is precedence for phenotypic differences in the ability of glucocorticoids to regulate gene expression in the brain. For example the corticotropin releasing hormone gene is repressed by glucocorticoids in the hypothalamic paraventricular nucleus, but upregulated in the central nucleus of the amygdala [25–27].

What may be the functional relevance of acute *Sgk1* mRNA upregulation in oligodendrocytes with acute stress and during the active portion of the diurnal sleep/wake cycle? In many cells types throughout the body *Sgk1* has low constitutive expression, but that expression can be rapidly increased in response to a wide range of cellular alterations often associated with cellular stress [8,28]. Our study, however, suggests that *Sgk1* expression plays a more central role to normal daily function of oligodendrocytes, as its levels are dynamically regulated by time of day and acute elevations of CORT that occur in response to brief stressful experiences. The daily increase in white matter *Sgk1* mRNA at the beginning of the circadian active phase indicates that upregulation of *Sgk1* expression in white matter is not necessarily a component of pathological processes [29].

There is growing appreciation that oligodendrocytes play a more active role in brain function than merely generating and maintaining axonal myelin sheaths. During brain development neural activity helps direct oligodendrocyte differentiation, proliferation, and axonal targeting [1]. In the adult brain there is recent compelling evidence for ongoing plasticity of myelination and activity-dependent alteration of oligodendrocyte function [3,30]. SGK1 is related in structure and function to some other kinases, such as PKA, PKC and PKB, that are pivotal in mediating intracellular adaptations to intercellular signals [5,31]. SGK1 in various cell types regulates the activity of a range of effector molecules including ion channels, membrane transporters, other intracellular signaling enzymes, and transcription factors [15]. In oligodendrocytes, SGK1 has been linked to a phospho-relay signaling pathway in which the protein is downstream from phosphatidylinositol 3-kinase (PI3K) and PI3K-3-phosphoinositide-dependent protein kinase (PDK1) and directly upstream of N-myc downstream-regulated gene 1 (NDRG1) [29]. Activation of NDRG1 subsequently leads to altered oligodendrocyte cell adhesion molecule expression and associated changes in cell morphology [4,29,32]. The regulation of *Sgk1* expression in oligodendrocytes by acute psychological stress and time of day may be an important component of a dynamic experience-dependent interplay between oligodendrocytes and neuronal function including neuroplasticity. If such is the case, then abnormalities in endogenous glucocorticoid secretion profiles [33,34] or glucocorticoid pharmacotherapy [35] may compromise this normal relationship.
